# Oral hygiene with age: A comprehensive study

**DOI:** 10.6026/9732063002001555

**Published:** 2024-11-30

**Authors:** Saba Anwar Kondkari, Sneha Masne Deshpande, Haritha Nimmagadda, Uttam Shetty, Sumeet Agarwal, Laresh Mistry, Prasad Mhaske, Pratibha Kavle

**Affiliations:** 1 Department of Dental Surgery, Bharati Vidyapeeth (Deemed to be University), Dental College and Hospital, Navi Mumbai, India; 2 Department of Oral Pathology and Microbiology, Bharati Vidyapeeth (Deemed to be University), Dental College and Hospital, Navi Mumbai, India; 3 Department of Medical Anatomy, Bharati Vidyapeeth (Deemed to be University), Dental College and Hospital, Navi Mumbai, India; 4 Department of Prosthodontics, Crown & Bridge and Implantology, Bharati Vidyapeeth (Deemed to be University) Dental College, Navi Mumbai, India; 5 Department of Paediatric and Preventive Dentistry, Bharati Vidyapeeth (Deemed to be University) Dental College, Navi Mumbai, India

**Keywords:** Oral hygiene, students, practices, knowledge, tooth-brushing

## Abstract

Dental health plays an important role in the overall well-being of children, their quality of life and performance at school. Majority of the children in India and other developing and underdeveloped nations are ignorant to the fact that oral health is considered as an important factor in overall health. The aim of this study was to conduct a survey to investigate the oral hygiene practices and differences among different age groups of School students in an associated school in Navi Mumbai. This cross-sectional study was carried out among students of ages 8 to 16 years, using a questionnaire that assessed their oral hygiene. A good oral hygiene practice was shown by maximum participants of middle age group.

## Background:

Dental health plays an important role in the overall well-being of children, their quality of life and performance at school [1]. Oral health is considered as an integral part of a child's overall health and influences their quality of life and lack of good oral hygiene results in a variety of dental health problems [2]. Healthy set of teeth and gums also form an important part of what is now known as facial aesthetics [3]. Oral diseases are considered as a public health problem because they lead to a number of non-dental related diseases too [4]. Loss of tooth is the most common effect of chronic oral diseases and is associated with physical, emotional and economic impacts [5]. Even the people living in cities fall prey to dental diseases due to their negligence in dietary habits and unhealthy lifestyles, despite having easy access to dental care [6]. Improper tooth brushing techniques, failure to carry out interdental cleaning and irregular dental visits results in dental plaque and calculus and plaque has been a significant factor in causing various oral diseases like periodontitis, gingival inflammation, *etc.* [7]. Translocation of periodontal microorganisms into the bloodstream and their further accumulation within atherosclerotic plaques, would contribute to enhance plaque instability and the risk of developing acute ischemic coronary events [8]. According to WHO estimates, around 3.5 billion people worldwide (or almost 50% of the population) suffer from some kind of oral disease [9]. The National Oral Health Survey, conducted in 2005, by the Indian Dental Association (IDA), highlighted that 95% of the population in India suffers from gum diseases, only 50% use a toothbrush and just 2% of the population visit the dentist [10].

Regarding the frequency and reason for the visit to the dentist, it was found that 35% of the Indian children never visited a dentist as compared to 11% of American children in the past 12 months [11]. Akila *et al.* (2019), conducted a study among 12 and 15-year-olds in Chennai, concluded that 12-year-olds had better oral hygiene practices than 15-year-olds and frequency of visiting dentists due to dental problems decreased with age 0 [12]. The aim of this study was to conduct a survey to investigate the oral hygiene practices among School students in an associated school in Navi Mumbai. The objectives of this study were to investigate the difference in hygiene practices among different age groups and to assess the knowledge of oral hygiene among the subjects. After reviewing the existing literature, there is an alarming need to provide oral health education among school children. Considering the aetiology of dental caries, there is a need to assess the host factors such as oral health knowledge, oral hygiene practices, dental visits and eating habits of the children. Hence a survey was conducted among the school children of various age groups, in Navi Mumbai region, to assess their oral hygiene habits, their knowledge and their oral hygiene practices.

## Methodology:

This cross-sectional study was carried out among students of ages 8 to 16 years. The students were asked (after gaining proper consent) to fill out a questionnaire which contained 21 questions that assessed their oral hygiene. The study was conducted for the duration of 2 months. The study was carried out after getting Institute's Ethical Committee approval and written consent from the study participants. This cross-sectional study was conducted in an associated school in Belapur region of Mumbai, assessing the oral health knowledge and practices among students. The school was selected at random after obtaining the list of private schools in Mumbai. This is a cross-sectional study which includes 200 students. The students were selected by convenience sampling. The subjects were stratified into 3 age groups, Age Group 1- Primary school students (Std- 3-4 / Age- 8-10), Age Group 2- Middle school students (Std- 5-8 / Age- 11-14) and Age Group 3- Secondary school students (Std- 9-10 / Age- 14-16). The questionnaire was designed by reviewing the literature and then modifying it according to local requirements. The questionnaire was designed on Google forms and a brief introduction to the study was given in the questionnaire itself. Face validation was done by different subject experts, those who were not included in the study and the suggestions were incorporated in the final questionnaire. It is a close-ended questionnaire which was distributed among the students via their respective class teachers. Consent of participation was obtained from parents of all children, only children with a parental consent were included in the study. Only students above the age 8 were included for better understanding of the questionnaire. The study population includes students of standards 3rd to 10th, of ages 8 to 16. Data obtained was compiled on a MS Office Excel Sheet (v 2019, Microsoft Redmond Campus, Redmond, Washington, United States). Data was subjected to statistical analysis using the Statistical package for social sciences (SPSS v 26.0, IBM). Descriptive statistics like frequencies and percentage for categorical data, Mean & SD for numerical data have been depicted. Comparison of frequencies of categories of variables with groups was done using chi square test.

## Results:

A total of 214 students filled the Google forms, of which 35 (16.4%) students belonged to age group 1 (*i.e.*, primary school students), 95 (44.4%) students belonged to age group 2 (*i.e.*, secondary school students) and 84 (39.3%) students belonged to age group 3 (*i.e.*, higher secondary school students) (Figure 1 - see PDF). Of the total students, 98 students (45.7%) were female, 116 students (54.2%) were male (Figure 2 - see PDF). The answers to various questions were assessed individually and also compared among the different age groups. The results of which are compiled in various tables and graphs below. (Figure 3 - see PDF), shows the comparative values, for the preferred tool and dentifrice for teeth cleaning, the type of brush used by the participants, the bristle type of the brush of various participants, the frequency of toothbrushing among the participants, the inclusion of tongue cleaning in tooth brushing routine, the tongue cleaning aid used by the participant, the duration of teeth cleaning, inclusion of flossing in their toothbrushing routines, the type of floss used by the participants, the frequency of flossing and use of fluoridated toothpaste. While, (Figure 4 - see PDF), shows the comparative values for, fluoridation of toothpaste, frequency of changing of toothbrush, way of buying tooth cleaning aids, inclusion of mouthwash, time interval between brushing and mouthwash, frequency of teeth decay, habit of rinsing mouth every time after eating, whether the participant considers oral health as important factor in overall health and frequency of dental check-up.

Most of the categories showed statistically non-significant data. On the question whether tongue cleaning was included while brushing, there was a statistically significant difference seen for the frequencies between the groups (p<0.05) with higher frequency for response yes with age group 2. Duration of brushing showed a statistically high significant difference for the frequencies between the groups (p<0.01) with higher frequency for response 1.5 to 2 minutes with age group 2. Whether flossing was included in oral hygiene routine, there was a statistically highly significant difference seen for the frequencies between the groups (p<0.01) with higher frequency for response No with age group 2. Also, frequency of flossing showed a statistically high significant difference for the frequencies between the groups (p<0.01) with higher frequency for response Not applicable with age group 2. Frequency of toothbrush showed a statistically high significant difference for the frequencies between the groups (p<0.01) with higher frequency for response Every 3 months with age group 2. Opinion on oral hygiene showed a statistically high significant difference for the frequencies between the groups (p<0.01) with higher free for response Yes with age group 2.

## Discussion:

Over the past years very few studies have been carried out regarding the oral health hygiene of school students in India. On the question about preferred tool for brushing or cleaning of teeth a total of 209 (97.7%) students of 214 claimed that they used a toothbrush as their preferred tool for teeth cleaning; also 4 students (1.9% overall) disclosed that they used neem (Azadirachta indica) stick for teeth cleaning and 1 student (0.5 % overall) used finger as a cleaning method. When questioned about the preferred material for teeth cleaning, 210 (98.1%) of the 214 students used toothpaste; 4 (1.9%) students of the overall 214 students used toothpowder. This is different from a study conducted by Amin *et al.* (2020), where the results showed that 81% of the children interviewed used a toothbrush and toothpaste to clean their teeth, 15% used their finger or neem stick with an abrasive such as toothpaste, charcoal, ash and salt [13]. Brushing of teeth at least twice a day is recommended, in the present study 22 students (57% overall) brushed their teeth twice a day, 87 of the students disclosed that they brush their teeth once a day, also, 2 students (0.9% overall) both from age group 3 brushed their teeth only when required and 3 students (1.4% overall) brushed their teeth only after meals, of which maximum students (*i.e.*, 65.7%) included in the age group 1. Answering the question about duration of tooth brushing 70 students (32.7%) brushed for 1 to 1.5 minutes, 110 students (51.4%) brushed for 1.5 to 2 minutes, 14 students (6.5%) brushed for 20 to 30 seconds and 14 students (6.5%) were not able to answer this question.

It is highly recommended to replace the toothbrush every 3 months to have maximum efficacy [14], here, 119 students (55.6%) change their toothbrush every 3 months, 30 students (14%) changed there's every 6 months, 59 students (27%) changed whenever required and 6 students (2.8%) were not aware about the frequency, of these the maximum responses were from age group 2 (66.3%), this can be compared with the study conducted by Md. Al-Amin *et al.* (2020), where 62% of the students were unaware of the frequency of changing toothbrushes [13]. Regarding the type of brush used; 207 (96.7%) of the 214 used a manual toothbrush, 7 students (3.3%) of the overall 214 used an electric toothbrush. In a study done by Shaima *et al.* [15], they concluded that electric toothbrushes are more effective than manual ones in plaque removal and in reducing the frequency of brush head replacement. Emine *et al.* [16] conducted a study to compare the role of different toothbrush bristle designs on cleaning efficacy and gingival recession, it was concluded that bristle design has little impact on plaque removal capacity of a toothbrush and any design of toothbrush bristle is safe enough to prevent gingival recession as long as soft bristle material is used, in the current study of the overall 214 participants, 4 students (1.9%) used a hard bristled toothbrush, 117 students (54.7%) of the overall 214 used a medium bristled toothbrush, 85 students (39.7%) of the overall 214 used a soft bristled toothbrush, also 8 students (3.7% overall) used very soft bristled toothbrush.

Regarding use of fluoridated toothpastes, 78 of the subjects (36.4%) used fluoridated toothpaste, 39 students (18.2%) used non-fluoridated toothpaste and 97 subjects (45.3%) were not aware if their toothpaste contains fluoride, this is similar to the study conducted by Jasmin (2015) where 36.8% of the subjects were well versed about the presence of fluoride in the toothpaste. But this result is highly different from the one conducted by Tay [17], where 91.4% participants used a fluoridated toothpaste. For 165 students (77.1%) parents and guardians bought the oral hygiene aid, 34 students (15.9%) bought their hygiene aids at random and 15 students (7%) bought after watching advertisement, this can be compared with the study conducted by Jasmin (2015) [15]. where almost half of the participants (49%) chose toothpaste on the advice of the dentist and about 182 were persuaded by brand advertisements. In our study tongue cleaning was reported by 141 students (65.9% overall), 25 (11.7%) students said they did not clean their tongue; 48 students (22.4% overall) said they cleaned their tongue sometime. 76 students (35.5%) of the 214 used a toothbrush for tongue cleaning, 100 students (46.7%) used a tongue cleaner and 32 (15%) did not use any aid, this is almost similar to the study conducted by Jasmin *et al.* in 2015 [18], where 82.8% participants agreed to tongue cleaning.

Regarding the flossing routine 82 students (38.3%) had never used floss, 37 students (17.3%) used floss sometimes, 38 students (17.8%) agreed to use a floss and 57 students (26.6%) had never heard of floss. When asked about frequency of flossing, 22 students (10.3%) flossed their teeth every time they brushed, 28 students (13.1%) used floss once a day. Further, 32 students (15%) used a regular dental floss, 26 students (12.1%) used an F/Y shaped floss pick and 17 students (7.9%) used water pick; this is very slightly different from a study conducted by Jasmin *et al.* (2015), regarding oral hygiene maintenance in children, where 14.6% of the subjects flossed their teeth. It also contraindicates a previous study conducted by Walsh [19] in San Francisco where 75% claimed to have used dental floss, at least once a day. This may be due to lack of awareness regarding cleaning of all teeth surfaces which also include using a dental floss. In the current study it was concluded that 112 students (52.3%) of the total 214 used a mouthwash regularly, 64 students (29.9) did not use a mouthwash, 38 students (17.8%) used a mouthwash sometimes, which is significantly better than the study conducted by Jasmin *et al.* [18]. where 35.1% participants used mouthwash. Also, about the time interval between brushing and mouth washing, 146 students (68.2%) directly used a mouthwash after brushing their teeth, 27 students (12.6%) used mouthwash 15 to 20 minutes after brushing. Regarding the frequency and visit to dentist 119 students (55.6%) visited a dentist only when suffering from a toothache, 53 students (24.8%) visited a dentist regularly and 42 students (19.6%) had never visited a dentist, as opposed to a study conducted in 2016 by Navneet *et al.* where 35% of the subjects have never visited a dentist [11]. Regarding the prevalence of caries, 28 students (13.1%) had dental caries more than once, 75 students (35%) never had caries in their teeth, 69 students (32.2%) had caries just once and 42 students (19.6%) were not able to answer this question, this can be compared with a study conducted by NIH where 27.9% subjects had dental caries from the ages 2 to 5 and 51.17% from the ages 6 to 11. Also, 81.8% of the participants considered oral health as an important factor in overall development of an individual.

## Conclusion:

A good oral hygiene practice was shown by maximum participants of age group 2 (Ages 8 to 10), this may be linked to good oral hygiene awareness among the students who are of around elder age and they are mature enough to understand that a good oral hygiene routine is beneficial for overall development of health. From the current findings it is evident that age group 1 (Ages 11 to 13) and 3 (Ages 14 to 16) showed a less good oral hygiene than age group 2. School based education and oral health awareness programmes are of a dire need.

## References

[1] Barbosa T.S, Gavião MBD (2008). Int J Dent Hyg..

[2] Tadin T (2022). Healthcare..

[3] Sharna N (2019). Dent J (Basel)..

[4] Batra P (2020). J Oral Biol Craniofac Res..

[5] Peres M.A (2019). The Lancet..

[6] Kapoor D (2014). Indian J Dent..

[7] Lertpimonchai A (2017). Int Dent J..

[8] Paul B (2014). J Family Med Prim Care..

[9] Jain N (2024). Oral Dis..

[10] Nocini R (2020). Blood Coagulation & Fibrinolysis..

[11] Grewal N, Kaur M (2007). Journal of Indian Society of Pedodontics and Preventive Dentistry..

[12] Ganesh A (2019). Journal of Indian Association of Public Health Dentistry..

[13] Bhuiyan M.A.A (2020). Dent J (Basel)..

[14] Warren P.R (2002). J Clin Dent..

[15] Bahammam S (2021). Int J Environ Res Public Health..

[16] Cifcibasi E (2014). Eur J Dent..

[17] Tay H.L (2009). Community Dent Health..

[18] Winnier J.J (2015). International Journal of Oral Health and Medical Research.

[19] Walsh M.M (1985). Community Dent Oral Epidemiol..

